# Immigrants’ Experiences of Maternity Care in Japan

**DOI:** 10.1007/s10900-013-9679-8

**Published:** 2013-04-23

**Authors:** Yukari Igarashi, Shigeko Horiuchi, Sarah E. Porter

**Affiliations:** 1 Maternal Infant Nursing and Midwifery, St. Luke’s College of Nursing, 10-1, Akashi-chou, Chuo-ku, Tokyo, 104-0044 Japan; 2 Oregon Health & Science University School of Nursing, 3181 S. W. Sam Jackson Park Road, Portland, OR 97239 USA; 3 46 Kaui Place, Kula, HI 96790 USA; 4 Maternal Infant Nursing and Midwifery, St. Luke’s College of Nursing, 10-1, Akashi-chou, Chuo-ku, Tokyo, 104-0044 Japan; 5 St. Luke’s Birth Clinic, 1-24, Akashi-chou, Chuo-ku, Tokyo, 104-0044 Japan

**Keywords:** Culturally diverse immigrant women, Maternity care, Japan, Literacy

## Abstract

Language and cultural differences can negatively impact immigrant women’s birth experience. However, little is known about their experiences in Japan’s highly homogenous culture. This cross-sectional study used survey data from a purposive sampling of immigrant women from 16 hospitals in several Japanese prefectures. Meeting the criteria and recruited to this study were 804 participants consisting of 236 immigrant women: Chinese (n = 83), Brazilian (n = 62), Filipino (n = 43), South Korean (n = 29) and from variety of English speaking nations (n = 19) and 568 Japanese women. The questionnaire was prepared in six languages: Japanese (kana syllables), Chinese, English, Korean, Portuguese, and Tagalog (Filipino). Associations among quality of maternity care, Japanese literacy level, loneliness and care satisfaction were explored using analysis of variance and multiple linear regression. The valid and reliable instruments used were Quality of Care for Pregnancy, Delivery and Postpartum Questionnaire, Rapid Estimate of Adult Literacy in Medicine Japanese version, the revised UCLA Loneliness Scale-Japanese version and Care satisfaction. Care was evaluated across prenatal, labor and delivery and post-partum periods. Immigrant women scored higher than Japanese women for both positive and negative aspects. When loneliness was strongly felt, care satisfaction was lower. Some competence of Japanese literacy was more likely to obstruct positive communication with healthcare providers, and was associated with loneliness. Immigrant women rated overall care as satisfactory. Japanese literacy decreased communication with healthcare providers, and was associated with loneliness presumably because some literacy unreasonably increased health care providers’ expectations of a higher level of communication.

## Introduction

Among the developed nations, Japan remains one of the most highly homogeneous cultures, or monoculture [1] as foreigners make up only about 1.5 % of the population [2]. Thus, the idea of becoming a “multicultural society” is a recent phenomenon [3, 4]. With the numbers of immigrant women who are marrying Japanese men increasing [5], cultural and language barriers may make it difficult for culturally diverse immigrant women (CDW) to receive necessary care because they are easily misunderstood [5, 6]. Despite their need of support and protection during birth, there is no widespread institutional endorsement of the need to provide culturally sensitive care.

Therefore, maternity care and research reflective of a multicultural society is in an early phase of development in Japan. Having an evaluation of maternity care from the perspective of CDW could contribute toward developing cultural awareness and sensitivity in maternity care in Japan.

## Background

### Midwifery Studies about Women from Culturally Diverse Backgrounds

Midwifery studies describing the unique birth experiences in a variety of cultures proposed that transcultural care was specific for each culture [7–11]. Interview-based studies from several countries revealed unique rituals surrounding birth, and birth experiences of immigrant women in host countries [12–16]. Given the uniqueness of cultures’ birthing experiences, nursing researchers have introduced the subject of culturally competent or transcultural nursing for the nursing curriculum, and also actual programs of culturally competence or transcultural nursing care [17].

### Midwifery Studies about Women from Culturally Diverse Backgrounds in Japan

There has been little clinical research or other types of scholarly investigations conducted related to the pregnancy, delivery or postpartum experiences of CDW in Japan. A few descriptive reports focused mainly on abnormal pregnancy conditions and delivery that were related to cultural and linguistic differences [18–20]. The concept of *tobikomi*-*bunben* (“no medical care during the gestational period, then rush to the hospital after contractions started”) was reported as a characteristic behavior of CDW [21, 22]. Earlier nursing research about CDW pregnant women began as case reports about nursing care problems or about issues, which concluded that ‘language differences’ were associated with negative health results [23–25]. As solutions for the language differences, the researchers proposed: ‘modifying the manner of communicating’, ‘the preparation and effective use of social resources’ and ‘co-operation with non-medical staff and communities that supported immigrants’. These solutions were commonly implied in similar surveys, but the results have yet to impact maternity care for CDW.

### Immigrant Women’s Expectations about Childbirth

It is widely accepted that women’s childbirth experiences are influenced by their culture [26]. Even so, various studies document that regardless of the woman’s culture, what she wants during childbirth is respect, warmth and support [27, 28]. When women migrate to another country and give birth, it is important for health care providers in the host country to provide culturally sensitive care. [26]. Sharts-Hopko [29] interviewed 20 American women giving birth in Japan. Data were analyzed within a stress-coping model and revealed that women had a sense of isolation. They needed security, a sense of control, affirmation and cultural support. Several large-scale studies [30, 31] of prenatal and birth care of immigrant women in Australia found that what they wanted was safe, kind, supportive, and respectful care. Women were less concerned that caregivers knew little about their cultural practices than they were about care they experienced as unkind, rushed, and unsupportive. Furthermore, cultural issues were not paramount during the postpartum hospitalization period; immigrant women’s concerns were about having more help with infant feeding and obtaining enough rest [28].

There were also a few research studies using interviews that explored CDW’s feelings during childbirth in Japan revealing their sense of loneliness [6, 32]. A pilot study [33] consisting of interviews conducted with nine CDW to explore and discover their childbirth experiences in Japan also found they had experienced loneliness. To deepen understanding of CDW’s experiences of giving birth in Japan a second pilot study by Fujiwara, in 2007 [34] employed an original questionnaire, developed from the first pilot study. The participants were 134 women, Japanese women (JW) (n = 103) and CDW (n = 31). Findings revealed that CDW tended to be more hesitant to talk with healthcare providers than JW. This result was associated with both language differences, and communication behaviors. To develop more culturally sensitive nursing care a more comprehensive approach was needed to evaluate maternity care from the perspective of CDW compared to Japanese women from the host country, Japan.

## Purpose

The primary purpose of this study was to analyze and compare evaluations from CDW and JP about the maternity care they received in Japan. The research question was: What are the associations among the quality of maternity care, loneliness, Japanese literacy level and care satisfaction for CDW.

## Methods

The research design was a cross-sectional descriptive study using survey data from a purposive sampling. Two groups, CDW and JW, evaluated their maternity care received in Japan during their pregnancy, delivery and postpartum periods. The selection of participants’ nationalities was based on the Ministry of Health, Labor and Welfare [35] analyses indicating the areas with their higher-ranking birth rates in Japan. Therefore, participants were recruited from 16 hospitals in several prefectures where a large number of foreigners were registered. Eligibility criteria were: (1) experienced pregnancy, delivery and post partum period in Japan; (2) experienced either vaginal or c-section delivery within the past two to four days; (3) understood one of the six languages used for the questionnaire; (4) consented to participate in this study. Exclusion criteria were: (1) gave birth after long-term hospitalization due to pregnancy complications; (2) was in poor physical condition or (3) had a premature infant in poor condition. Meeting the criteria and recruited to this study were a total of 804 participants; 236 CDW: Chinese (n = 83), Brazilian (n = 62), Filipino (n = 43), South Korean (n = 29) and women from other countries, including a variety of English speaking nations (n = 19) and 568 Japanese women. Thus the questionnaire was prepared in six languages: Japanese (with kana syllables), Chinese, English, Korean, Portuguese, and Tagalog (Filipino). Ten copies of the questionnaire in each language were prepared. A total of 60 questionnaires for CDW, and 40 questionnaires for the Japanese questionnaire for JW, were given to each of the 16 hospitals agreeing to participate in the study yielding a total distribution of 1,600 self-administered questionnaires.

St. Luke’s College of Nursing Ethics Research Committee granted study approval. The ethics committee of each hospital also approved the study. The researcher explained the objective and significance of this study, procedure, and ethical considerations to the healthcare staff to gain cooperation. Following the ethics committees’ approval data collection commenced, from March 1, 2008 to September 30, 2008 and was collected once a month by post or visiting hospitals. Return of the questionnaire was considered as consent to participate. Hospital staff assured participants of their right to refuse to participate and that their responses would be anonymous. The researcher regularly called or visited each hospital to maintain the quality of the research condition and to discuss the study with healthcare staff that were responsible for distributing the questionnaire to eligible participants.

### Measures

#### Respect, Understand and Cold

Quality of Care for Pregnancy, Delivery and Postpartum Questionnaire (QCQ), which was an original investigator developed measurement, was employed to measure maternity care quality. The measurement was based on Donabedian’s three variables for evaluating the quality of medical care: setting, process and outcome [36, 37]. In this study, *setting* was organization or preparation in hospitals and nursing managing system, *process* was the way of providing care and care attitude of healthcare providers and *outcome* was women’s behavior and changes in feeling. QCQ was revised from Fujiwara’s unpublished 2010 pilot study II and was based on the results of previous studies [6, 33]. QCQ, a self-completed questionnaire of 103 items, consisted of three factors: (1) *Respect* (feeling respected by health care provider and trusting the health care provider); (2) *Understanding* (explanations by healthcare givers that were supportive and led to the respondents feeling of being more understood by the healthcare giver); and (3) *Cold* (distant and unhelpful attitude of the healthcare giver). The QCQ was measured during three time-periods: pregnancy (31 items), labor and delivery (35 items) and postpartum (37 items). The ordinal level responses, on a 5-point Likert scale, ranged from 5 = *strongly agree* to 1 = *strongly disagree.* Thus the lowest to highest QCQ scores possible were: pregnancy (31–155), labor and delivery (35–175) and postpartum (37–185). The highest combined score possible was 515. Cronbach’s α was greater than 0.80 for *each factor* except for *Cold* in pregnancy (α = 0.50); whereas, Cronbach’s α was greater than 0.90 for each time-period.

#### Care Satisfaction

This measure consisted of one item, *Care satisfaction*, and was measured across the three time-periods by a visual analogue scale (VAS). The responses ranged from 10 = *extremely satisfied* to 0 = *extremely dissatisfied*.

#### Loneliness

The revised UCLA Loneliness Scale-Japanese version was employed to measure Loneliness while receiving healthcare services during the three periods. Russell [38, 39] created the UCLA Loneliness Scale based on his understanding that loneliness occurs from conditional perspectives, and that loneliness is a unitary dimension. This scale was translated and revised by Moroi [40], and then named the Japanese revised UCLA Loneliness Scale. The subjects for this scale were from teenagers to elderly people, which meant it applied to a wide age of people. There are 20 items in the scale including ten reversed items. The revisions for this study included changing the word ‘people’ to ‘healthcare staff’ and modifying the response from a 4-point to a 5-point Likert scale: 5 = *very strongly agree* to 1 = *absolutely disagree.* Therefore the possible score range for this study was 100–20. These modifications were recommended and permitted by Dr. Katsuhide Moroi, (personal communication November 28, 2007) creator of the Japanese-revised UCLA Loneliness Scale.

#### Literacy Level

The Rapid Estimate of Adult Literacy in Medicine (REALM) is a widely used 66—item assessment of medical literacy with established reliability and validity. This test was recognized in 1991, and in use since 1993 [41]. The REALM Japanese version (REALM J-v) is a medical word recognition and pronunciation assessment using a 30-item questionnaire based on the results of the Fujiwara’s pilot study [34]. The score (1 = *pronounced correctly;* 0 = *pronounced incorrectly*) ranged from a perfect score of 30–0. Approval for translation into Japanese was obtained from Dr. Terry Davis, (personal communication: February 2, 2007) who developed REALM. The REALM J-v was only used to measure CDW’s literacy level of Japanese medical terms.

Data were analyzed using SPSS ver. 15.0. T-tests were employed to compare all items between CDW and JW in the pregnancy, delivery and postpartum period. Three factors of QCQ; Respect, Understanding and Cold, the Literacy Level, Loneliness and Care Satisfaction over the three time-periods, were analyzed using multiple regression analysis to examine associations and differences between CDW and JW.

## Results

The majority of the 236 CDW had resided in Japan less than 15 years; two Chinese women had resided in Japan over 15 years. The majority (79 %) of the CDW and JW (80 %) delivered vaginally. Leaving 21 % of CDW and 20 % of JW having cesarean sections. No significant differences were found between CDW and JW for c-sections. However, CDW had a higher proportion (48.0 %; 24/50) than JW (40.7 %, 46/113) of emergency c-sections. Within that group the CDW-Chinese woman showed a higher proportion of emergency c-sections (61.1 %; 11/18) (See Table 1).

**Table 1:** Characteristic of participants by nationality and maternity history

Characteristic	Total	JW (Japanese)		CDW (immigrant)		Nationalities									
						Chinese		South Korean		Filipino		Brazilian		Others	
	N	n	(%)	n	(%)	n	(%)	n	(%)	n	(%)	n	(%)	n	(%)
Nationality	804	568	(70.6)	236	(29.4)	83	(35.2)	29	(12.3)	43	(18.2)	62	(26.3)	19	(8.0)
Maternal age (year)			30.7 ± 4.9		29.2 ± 5.3		29.6 ± 4.9		31.4 ± 3.6		30.1 ± 5.3		26.4 ± 5.7		31.1 ± 3.8
Time in Japan (year)					6.3 ± 4.5		6.2 ± 3.7		6.4 ± 4.2		6.6 ± 6.8		6.3 ± 3.7		5.2 ± 4.7
Parity															
Primipara	444	302	(53.2)	142	(60.2)	60	(72.3)	20	(69.0)	13	(30.2)	36	(58.1)	12	(63.2)
Multipara	360	266	(46.8)	94	(39.8)	23	(27.7)	9	(31.0)	30	(69.8)	26	(41.9)	7	(36.8)
Method of delivery															
Vaginal delivery	641	455	(80.1)	186	(78.8)	65	(78.3)	23	(79.3)	32	(74.4)	53	(85.4)	13	(68.4)
Cesarean section	163	113	(19.8)	50	(21.2)	18	(21.7)	6	(20.7)	11	(25.6)	9	(14.5)	6	(31.5)
Elective	93	67	(59.3)	26	(52.0)	7	(38.9)	2	(33.3)	5	(45.5)	6	(66.7)	6	(100.0)
Emergency	70	46	(40.7)	24	(48.0)	11	(61.1)	4	(66.7)	6	(54.5)	3	(33.3)	0	(0.0)

### Comparison of QCQ and Care Satisfaction

Comparing the items’ scores of the QCQ, CDW consistently scored higher on all three factors than JW throughout the three time-periods. In particular, CDW scored higher than JW on both positive questions such as relating to ‘respect and faith’ and negative questions such as ‘not allowed to ask questions’, ‘midwives avoided me’ and ‘cold attitude’. However, the mean score of Care satisfaction for CDW was 8.8 points, indicating totally satisfied, and was a slightly higher score than JW (*M* = 8.4).

### Comparison of Associations of Factors and Nationalities

Comparisons of the three QCQ factors: *Respect*, *Understanding* and *Cold* and the *Care satisfaction* factor across the three time-periods were conducted using analysis of variance and Tukey’s HSD multiple comparison Test (See Table 2). The pregnancy and postpartum periods had similar results, displayed in Table 2. Brazilian participants had the highest mean score for the factor *Respect* across all three periods, which was significantly different from the other five nationalities. Interestingly, even though Brazilians rated *Cold,* a negative factor, they also rated *Care satisfaction* the highest.

**Table 2:**
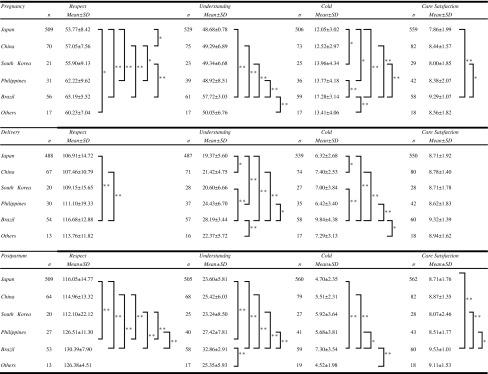
Comparison of quality of care associations and nationalities by periods (multiple comparison; Tukey’s HSD test)

* *p* < 0.05; ** *p* < 0.01

### Loneliness

Filipino participants had the highest mean score (*M* = 42.5 ± 11.9), which indicated marked *Loneliness*. The mean scores in descending order by immigrant nationality: South Korea (*M* = 41.07 ± 12.7), Brazil (*M* = 40.62 ± 11.7), and China (*M* = 38.63 ± 10.4) (Table 3). Women from Japan (*M* = 36.98 ± 10.8) and the Philippines, in particular, had a significant difference (*p* < 0.024).

**Table 3:** Comparison of mean loneliness scores by nationality

Nationality	Number of subjects	Loneliness score mean	SD	Minimum	Maximum
Japan	568	36.98	10.783	20	66
China	83	38.63	10.371	20	61
South Korea	29	41.07	12.663	23	81
Philippine	43	42.49	11.858	22	65
Brazil	62	40.62	11.691	20	66
Others	19	35.53	11.162	23	57
Total	804	37.82	11.042	20	81

### Literacy Level

The highest score possible for the *Literacy level* was 30. The mean score was 17.36 ± 10.82 for all CDW. The mean score of each nationality in descending order was: Chinese (19.57 ± 10.15), South Korean (16.72 ± 11.69), Filipino (0.84 ± 1.75) and Brazilian (0.37 ± 0.91).

### Loneliness and Literacy Level Effects on Maternity Care Evaluations by CDW and JW

Table 4 presents the results of the simple regression analysis for both CDW and JW. The analyses used the three QCQ factors; *Respect*, *Understanding* and *Cold*, and the *Care satisfaction* as dependent variables and *Loneliness* as the independent for both CDW and JW across the three periods. *Literacy level* was also an independent variable for CDW. There were 12 results from the analysis in each group. For CDW (n = 236), *Loneliness* showed a correlation coefficient range from 0.22 to 0.48 (*M* = 0.34). Significant correlations were found in all factors (*p* < 0.01). In each of the three periods, *Respect* and *Loneliness* showed strong negative correlations, and this trend was the strongest in the postpartum period. There were strong negative correlations of *Literacy level,* with *Respect* in the pregnancy period, and with *Understanding* in the delivery and postpartum period. This trend was the strongest in the delivery period.

**Table 4:** Loneliness effects on childbirth care evaluations by Immigrant and Japanese women across three time periods

Immigrant women n = 236 (Independent variables: Loneliness and Literacy level)				
Period		Pregnancy		
Dependent variable	Respect	Understanding	Cold	Care satisfaction
Correlation with loneliness	−.331	−.249	.222	−.396
*t*	−4.628	−3.565	3.120	−6.199
Correlation with literacy level	−.397	−.349	.459	−.087
*t*	−5.744	−5.206	7.137	−1.270

Period		Delivery		
Dependent variable	Respect	Understanding	Cold	Care satisfaction
Correlation with loneliness	−.349	−.273	.348	−.420
*t*	−4.822	−3.902	5.125	−6.641
Correlation with literacy level	−.194	−.477	.220	−.105
*t*	−2.571	−7.507	−3.132	−1.517

Period		Postpartum		
Dependent variable	Respect	Understanding	Cold	Care satisfaction
Correlation with loneliness	−.402	−.259	.387	−.438
*t*	−5.548	−3.669	5.969	−7.967
Correlation with literacy level	−.353	−.425	.209	−.107
*t*	−4.809	−6.461	3.053	−1.569

Japanese women n = 568 (Independent variables: Loneliness)				
Period		Pregnancy		
Dependent variable	Respect	Understanding	Cold	Care satisfaction
Correlation with loneliness	−.569	−.406	.268	−.445
*t*	−15.590	−10.193	6.239	−11.721

Period		Delivery		
Dependent Variable	Respect	Understanding	Cold	Care Satisfaction
Correlation with Loneliness	−.573	−.252	.546	−.404
*t*	−15.431	−5.737	15.115	−10.344

Period		Postpartum		
Dependent variable	Respect	Understanding	Cold	Care satisfaction
Correlation with loneliness	−.674	−.276	.563	−.467
*t*	−20.546	−6.428	16.075	−12.488

All items were statistically significant (*p* < 0.01)

For JW (n = 568) the correlation coefficient ranged from 0.25 to 0.67 (*M* = 0.46). Significant correlations were found in all factors (*p* < 0.01). In each of the three time-periods, *Respect* and *Loneliness* showed a strong negative correlation, and this trend was the strongest in the postpartum period just as it was for CDW. Likewise, *Care satisfaction* showed a negative correlation with *Loneliness*, which was the strongest in the postpartum period as well.

### Associations among QCQ, Literacy Level and Loneliness in CDW in the Sub-group of Chinese and South Koreans

Filipinos (0.84 ± 1.75) and Brazilians (0.37 ± 0.91) had very low scores on the REALM J-v; hence, it was difficult to examine the association with Literacy level and other factors. Therefore, only the sub-group of Chinese and South Koreans (*n* = 112) was used for this analysis as they had higher scores on the REALM J-v. Table 5 displays the results of the multiple regression analysis using dependent variables: three factors of QCQ (*Respect*, *Understanding* and *Cold*) and *Care satisfaction*. The independent variables were: *Loneliness* and *Literacy level*. The multiple correlation coefficient (*R)* ranged from 0.42 to 0.66 (M = 0.52). Significant correlations were found in all factors (*p* < 0.01) except the association with *Loneliness* and *Cold* in the pregnancy period, according to the *R* and *t* value. *Literacy level* also had significant associations with six of the 12 factors. *Care satisfaction* in the three periods, *Loneliness* and *Literacy level* were negatively correlated, and *Loneliness* significantly affected *Care satisfaction.*

**Table 5:** Associations among care satisfaction, REALM Japanese version and Loneliness in CDW (sub-group of Chinese and South Korean) n = 112

Term	Dependent variables	*R*	Independent variables	*t*	*p* value
Pregnancy	Care satisfaction	0.552	REALM J-v Loneliness	0.019	0.985
				−6.752	0.000**
Delivery	Care satisfaction	0.609	REALM J-v Loneliness	−2.418	0.017*
				−7.8	0.000**
Postpartum	Care satisfaction	0.656	REALM J-v Loneliness	−1.589	0.115
				−8.999	0.000**

** *p* < 0.01; * *p* < 0.05

## Discussion

### Cultural Differences in Evaluating Quality of Maternity Care

Compared with previous studies [30, 42, 43] about childbirth experiences of immigrant women, overall care of respondents in this study was satisfactory, even though Yelland et al. [28] did report that immigrant women also had negative experiences with healthcare providers because of cultural differences and hospital routines. When we considered care evaluated by women, it was important to not only focus on their satisfaction, but also on their ratings of many aspects of care and the association with care satisfaction and aspects of care.

CDW and JW responded in different ways regarding *Care satisfaction* and one likely explanation is because of their cultural differences. Their evaluating tendencies matched what theorists noted about cultural characteristics in each nationality [44]. The result of the *Loneliness scale* was remarkable in that Filipino women marked the highest score of all nationalities; they evidenced the most loneliness of all the respondents. Because of Filipino’s cultural communication, they are most likely to value kinship and interpersonal relationship, and sharing identity with others [44, 45]. They would likely feel a sense of loneliness if their space and relationships were limited in hospitals’ situations and they felt far removed from their country.

Brazilian women scored the highest in both negative and positive items, compared with factor scores by other nationalities. Brazilians commonly assessed ‘great’ in all matters. The reason for the highest score for the positive items was that it was influenced by a more effusive evaluation for good things, possibly based on what Purnell [46] referred to as their ‘cultural characteristic’. On the other hand, we also postulate that the reason for the lowest evaluation for negative items was influenced by their different communication patterns from Japanese. Brazilians use touch, eye contact and a ‘kiss on the cheek’ in every day communication [46]. They open communication with feelings, speak informally and close personal space is common. This is totally different from the Japanese communication style. Japanese respect others by keeping space and building interpersonal relationships which do not include touching and kissing [47]. While both Japanese and Brazilians cultures are characterized by what Edward Hall [48] called ‘high context’, relying on contextual rather than verbal cues, Brazilians might be confused or even feel shunned by such communications with Japanese; this could have been reflected in their evaluation of childbirth care.

Chinese and South Korean participants showed similar evaluations as Japanese. Asians are thought to have defensive attitudes for childbirth compared with other nationalities [49]. Therefore it is thought that they are likely to rate items lower than others. However, Chinese seemed to rate items both higher and lower compared to Japanese and South Korean. Japanese and South Korean cultures value the balance between person and others [50] hence, these nationalities might have similar score ratings such as lower evaluations. China is located geographically in Asia, yet it has different cultural characteristics from Japan and South Korea, which might be reflected in their evaluation. Wang [51] explained that Chinese cultural communication is characterized by their dislike of touching and face-to-face communication; in particular, direct eye contact makes them uncomfortable. Wang [51] therefore recommended that healthcare providers sit next to patients to enhance positive communication. These reasons might have influenced their evaluation producing a wider variation in results especially for the items of ‘respect and trust’.

### Feelings and Emotion Beyond Verbal Communication

The literacy level was used to examine the association between Japanese literacy level and evaluation of quality of maternity care. Participants originating from China and South Korea scored much higher than participants from the Philippines and Brazil. Table 5 shows the Loneliness factor and the literacy level in relation to other factors. ‘Understanding’, ‘Literacy level’ and ‘Loneliness’ showed negative correlations. This suggested that high competence of Japanese literacy, strong loneliness, and insufficient ‘Understanding’ (explanations with supporting ways leading to greater understanding), were associated with, and affected evaluations of childbirth care. This result was similar to the study of Fujiwara et al. [33], which reported that CDW were more likely to receive insufficient explanations from healthcare providers if they could understand ‘everyday Japanese’ a little, because then healthcare providers’ would over estimation CDW’s literacy level. In these cases, healthcare providers generally talked to them with medical terms beyond their comprehension, thus CDW were confused and began to feel isolated and lonely. Similarly, this study identified that Japanese literacy sometimes obstructed positive communications between CDW and healthcare providers, and induced loneliness while in the healthcare setting.

### Implications for Practice and Further Study

Healthcare providers need to understand that regardless of CDW’s or JW’s background, all women wish to experience satisfying maternity care. Women value ‘respect and trust’ when receiving maternity care. CDW need the same quality of care and relationship as JW. Therefore, becoming focused on only language and cultural differences in care is not always the main strategy. Healthcare providers also need to understand that their own caring behavior and attitudes can have a positive or negative impact on women from other cultures. When possible healthcare providers need to adapt their communication style to be more attune to the culture of CDW [26].

For future studies, the QCQ needs to be improved as a generalized measurement for the evaluation of the quality of maternity care. REALM J-v also needs to be improved for use as a common screening test of Japanese literacy level in clinical settings. It is also possible that systematic subtle discrimination occurred by the health care provider thus influencing the respondents’ experience. This possibility needs exploring. Educational programs on cultural competence must be developed for healthcare providers, and medical and nursing students to inform them of the implications for care of culturally diverse patients.

## Limitations

Minor errors may have occurred in the comparison of nationalities because the representation of participants was not a random sample. A purposive non-probably sampling was employed for recruiting CDW who were living the Kanto, Toukai and Kansai regions where there are more CDW than in other regions. Therefore inclusion of a broader sample could lead to wider variations. The REALM J-v may have been a difficult test for Filipino and Brazilian respondents because of their lack of fluency with kanji characters. The degree of difficulty could be different depend on not only nationality but also level of education and cognitive ability; hence the measurement of literacy level produced minor errors.

## Conclusion

While CDW rated overall care as satisfactory, several care items were rated unsatisfactory and depended on the aspects of care. This evaluation could be affected by cultural differences. Brazilians scored both positive and negative items of QCQ, with wider variation than the other groups and Filipinos showed more extreme loneliness ratings based on different cultural communication styles. South Korean and Japanese participants tended to have similar scores, yet Chinese participants were more likely to provide more extreme ratings, that is, both higher and lower scoring. Japanese literacy level and evaluation of maternity care revealed that competence of Japanese literacy was more likely to obstruct positive communication between CDW and healthcare providers, and induced loneliness while in the healthcare setting. Similarly, when loneliness was strongly felt, care satisfaction was rated low.
